# Genome Sequences of Cluster K Mycobacteriophages DrHayes, Urkel, and SamuelLPlaqson

**DOI:** 10.1128/genomeA.01388-16

**Published:** 2017-04-20

**Authors:** Kirk R. Anders, Alex M. Murphy, William F. Ettinger, Douglas Kempthorne, Charles Kittridge, Alex Kures, Sarah Lundgren, Jacob Masters, Rachel Noyes, Christina Winters, Perry Yazzolino, Kasandra Ziebert, Joseph Haydock, Stephen Hayes, Rebecca A. Garlena, Daniel A. Russell, Marianne K. Poxleitner, Ann-Scott H. Ettinger

**Affiliations:** Department of Biology, Gonzaga University, Spokane, Washington, USA

## Abstract

Mycobacteriophages DrHayes, Urkel, and SamuelLPlaqson were isolated from soil samples in Spokane, WA, using *Mycobacterium smegmatis* mc^2^155 grown at room temperature. The three genomes differ by only a few nucleotides, are 60,526 bp long, have 97 predicted protein-coding genes and one tRNA gene, and are members of subcluster K1.

## GENOME ANNOUNCEMENT

Bacteriophages are the most numerous life form on the planet (1). The genomic sequences of phages isolated on *Mycobacterium smegmatis* mc^2^155 reveal an astounding genetic variation that provides insight into the diversity and evolution of these viruses (2, 3). We report here the genome sequences of three mycobacteriophages that were isolated and analyzed by undergraduate students in the introductory biology and genetics lab courses as part of the Science Education Alliance-Phage Hunters Advancing Genomics and Evolutionary Science (SEA-PHAGES) research and education program (4–6).

Phages DrHayes, Urkel, and SamuelLPlaqson were isolated from soil samples from different locations in Spokane, WA, following enrichment in liquid cultures of *M. smegmatis* mc^2^155. The phages were purified and amplified at room temperature (20 to 25°C). DrHayes and SamuelLPlaqson form turbid plaques, whereas Urkel forms clear plaques. Electron microscopy showed that each phage exhibits a siphoviral morphology with an isometric head. Purified DNA was sequenced by Illumina MiSeq using 140-bp single-end reads. Newbler assembled the reads into contigs with average coverages of 114- to 230-fold. The nearly identical genomes are 60,526 bp and have 66.2% G+C content, and each contains 3′ single-strand extensions with the sequence 5′-CTCGTAGGCAT-3′. Urkel carries four 1-bp substitutions compared to DrHayes and SamuelLPlaqson, and SamuelLPlaqson carries an additional substitution compared to the others. The genomes are most closely related to CrimD (93% nucleotide identity spanning 93% of genome lengths) and are members of subcluster K1.

Initial annotations were generated by Glimmer (7), GeneMark (8), Aragorn (9), and tRNAscan-SE (10) and manually refined to identify 97 protein-coding genes and one tRNA (tRNA^trp^) in each genome. Functions were predicted for 41 genes using BLASTP, the Conserved Domain Database at NCBI (11), and HHPred (12). The genome structures match the other K1 genomes closely: genes encoding virion assembly and structure proteins, lysis proteins, and host integration/excision proteins are located on the left arm and, although gene functions on the right arm are mostly unknown, several genes encode proteins related to nucleic acid metabolism, such as exonucleases, DNA primase, RusA resolvase, and RtcB RNA ligase (13). Interestingly, Urkel carries an R55G missense mutation in the immunity repressor gp44, which correlates with its clear plaque morphology.

The three newly isolated phages differ from CrimD with at least 11 mosaic differences, including gene substitutions, insertions, and deletions. There are three instances (genes *4*, *33*, and *86*) in which genes are conserved among the phages but are more highly diverged (45, 42, and 49% amino acid [aa] identity, respectively) than their flanking genes (73 to 95% aa identity); the functions of these are unknown. Also of note, gene *26*, which is situated within a syntenic block of putative tail protein genes, is unrelated to CrimD gene *26*. In fact, out of all 1,000+ sequenced mycobacteriophages, gene *26* is unique to DrHayes, Urkel, and SamuelLPlaqson. Finally, there are four CrimD genes (*58*, *64*, *69*, and *73*) that are absent from DrHayes, Urkel, and SamuelLPlaqson and thus are likely nonessential for lytic growth.

### Accession number(s).

The DrHayes, Urkel, and SamuelLPlaqson genomes are available at GenBank under the accession numbers KX657795, KX657796, and KX657794, respectively.
